# Restoration of the trochlear peaks is unnecessary with a kinematic alignment‐optimized femoral component as under‐stuffing results in equivalent or better patient‐reported outcome scores

**DOI:** 10.1002/ksa.12777

**Published:** 2025-07-07

**Authors:** Stephen M. Howell, Ahmed Zabiba, Patrick Sadoghi, Alexander J. Nedopil, Maury L. Hull

**Affiliations:** ^1^ Department of Biomedical Engineering University of California Davis California USA; ^2^ University of California at Davis School of Medicine Sacramento California USA; ^3^ Department of Orthopedics and Trauma Medical University of Graz Graz Austria; ^4^ Orthopädische Klinik König‐Ludwig‐Haus Lehrstuhl für Orthopädie der Universität Würzburg Würzburg Germany; ^5^ Department of Orthopedic Surgery University of California Davis California USA; ^6^ Department of Mechanical Engineering University of California Davis California USA

**Keywords:** Forgotten Joint Score, kinematic alignment, over‐stuffing, patellofemoral kinematics, prosthetic trochlear groove, trochlear peak height

## Abstract

**Purpose:**

Kinematic alignment (KA) total knee arthroplasty (TKA) aligns the femoral component to restore the pre‐arthritic posterior joint line, potentially altering the heights of the medial and lateral trochlear peaks. It remains unclear whether the femoral component should be adjusted to correct deviations in peak height. This study assessed whether >2 mm of under‐ or over‐stuffing in peak height negatively impacted patient‐reported outcome (PRO) scores compared to restoration within ±2 mm.

**Methods:**

The study included 115 KA TKAs performed with a KA‐optimized femoral component featuring a trochlea with a lateral ridge opening that creates a 20° valgus trochlear groove and a flattened medial ridge, and PROs at a mean of 22 (12–28) months. The surgeon measured the height of the trochlear peaks on the anterior femoral resection.

**Results:**

Peak under‐stuffing >2 mm occurred medially in 66% and laterally in 43%. Over‐stuffing >2 mm was too infrequent for statistical analysis. Compared to restoration within ±2 mm, medial under‐stuffing resulted in a non‐equivalent 6‐point higher Forgotten Joint Score (FJS) (*p* = 0.1087) and equivalent but 9‐ and 3‐point higher Knee Injury and Osteoarthritis Outcome Score for Joint Replacement (KOOS JR) (*p* < 0.0001) and Oxford Knee Score (OKS) (*p* = 0.0020). Lateral under‐stuffing yielded equivalent but 6‐, 12‐ and 3‐point higher FJS (*p* = 0.0484), KOOS JR (*p* < 0.0001) and OKS (*p* < 0.0001).

**Conclusion:**

The KA‐optimized femoral component features a patient‐specific trochlea that addresses anterior arthritic trochlear variations, which are reported to range from −24° varus to 30° valgus. One possible explanation for why >2 mm of under‐stuffing leads to superior PROs is that reducing the peak height compensates for over‐stuffing above the native trochlea caused by the prosthesis's proximal overreach, with a reported average of 17 mm.

**Level of Evidence:**

Level III.

**Abbreviations**
FJS: Forgotten Joint Score
KA: kinematic alignment
KA TKA: kinematically aligned total knee arthroplasty
OKS: Oxford Knee Score
SD: standard deviation
TKA: total knee arthroplasty

## INTRODUCTION

In total knee arthroplasty (TKA), some patients still report lower‐than‐expected patient‐reported outcome (PRO) scores. It is hypothesized that restoring the anterior compartment may enhance PROs [22, 37, 45].

**Figure 1:**
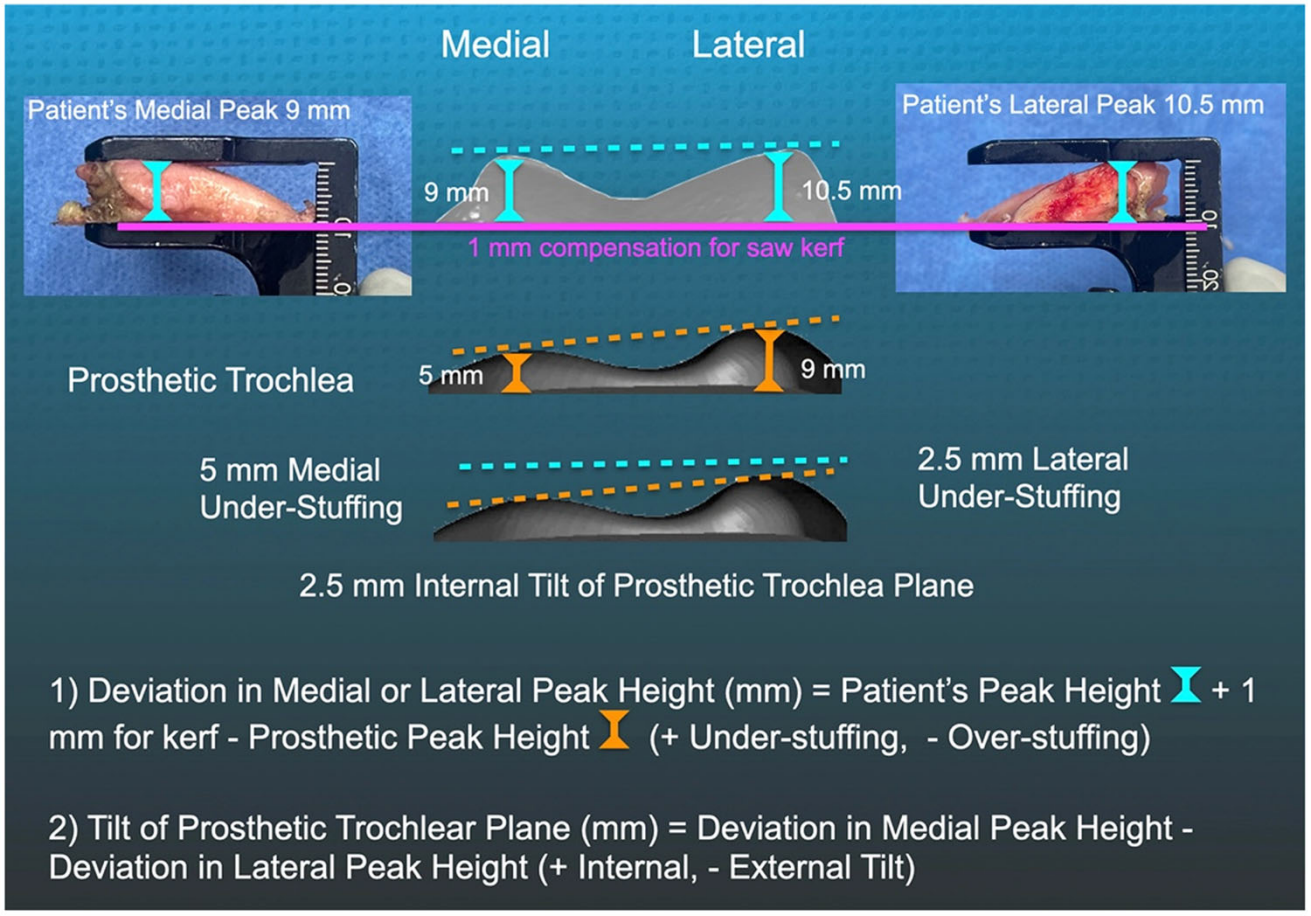
Schematics of the axial view of the left knee illustrate calliper measurements of 9 and 10.5 mm for the heights of the medial and lateral trochlear peaks of the patient's anterior femoral resection (top row). The second row shows the axial view of the prosthetic trochlea, which has a 5 mm medial peak height and a 9 mm lateral peak height that vary with the size of the femoral component and were provided by the manufacturer. The third row indicates the results obtained using the two equations, which calculated a 5 mm deviation in medial peak height and a 2.5 mm deviation in lateral peak height, resulting in under‐stuffing that caused a 2.5 mm internal tilt of the prosthetic trochlear plane.

On the femoral side, aligning the medial and lateral trochlear peaks and the internal–external (I–E) tilt of the prosthetic and patient trochlea helps restore the anterior compartment (Figure 1) [2, 22, 25, 40]. However, there is a limit to correcting medial peak under‐stuffing, as >1 mm posterior femoral over‐resection relative to the pre‐arthritic articular surface worsens the Forgotten Joint Score (FJS) by 34 points and the Oxford Knee Score (OKS) by 7 points [39].

Restoring the anterior compartment on the patellar side poses challenges due to difficulties in estimating the defect thickness caused by worn patellar cartilage and bone. Additionally, variations in the thickness of the patellar remnant can arise from the surgical technique used.

The freehand flush resection technique involves cutting the patella parallel to the posterior surface of the extensor mechanism. It is preferred over using a cutting guide because the patella remnant is more symmetrical [3, 4]. This approach allows for the thinnest possible patella remnant while preserving the tendinous attachments. In 90% of TKAs, the thickness of the patella‐implant construct is within ±1 mm of the intraoperative patella thickness [27]. Those who prefer the calliper resection technique should be aware that it is less accurate than the freehand flush technique, as the resurfaced patella is either thicker (up to 12 mm) or thinner (up to 7 mm) than the intraoperative patella in thickness in 44% and 28% of TKAs, respectively [13].

Kinematic alignment (KA), which resurfaces the pre‐arthritic knee, restores the native trochlea more effectively than functional alignment (FA), mechanical alignment (MA), gap‐balancing (GB), restricted KA (rKA) and inverse KA (iKA) [23, 24, 36, 37]. However, KA still has some outliers in trochlear orientation, with the potential for unknown adverse clinical consequences [24].

**Figure 2:**
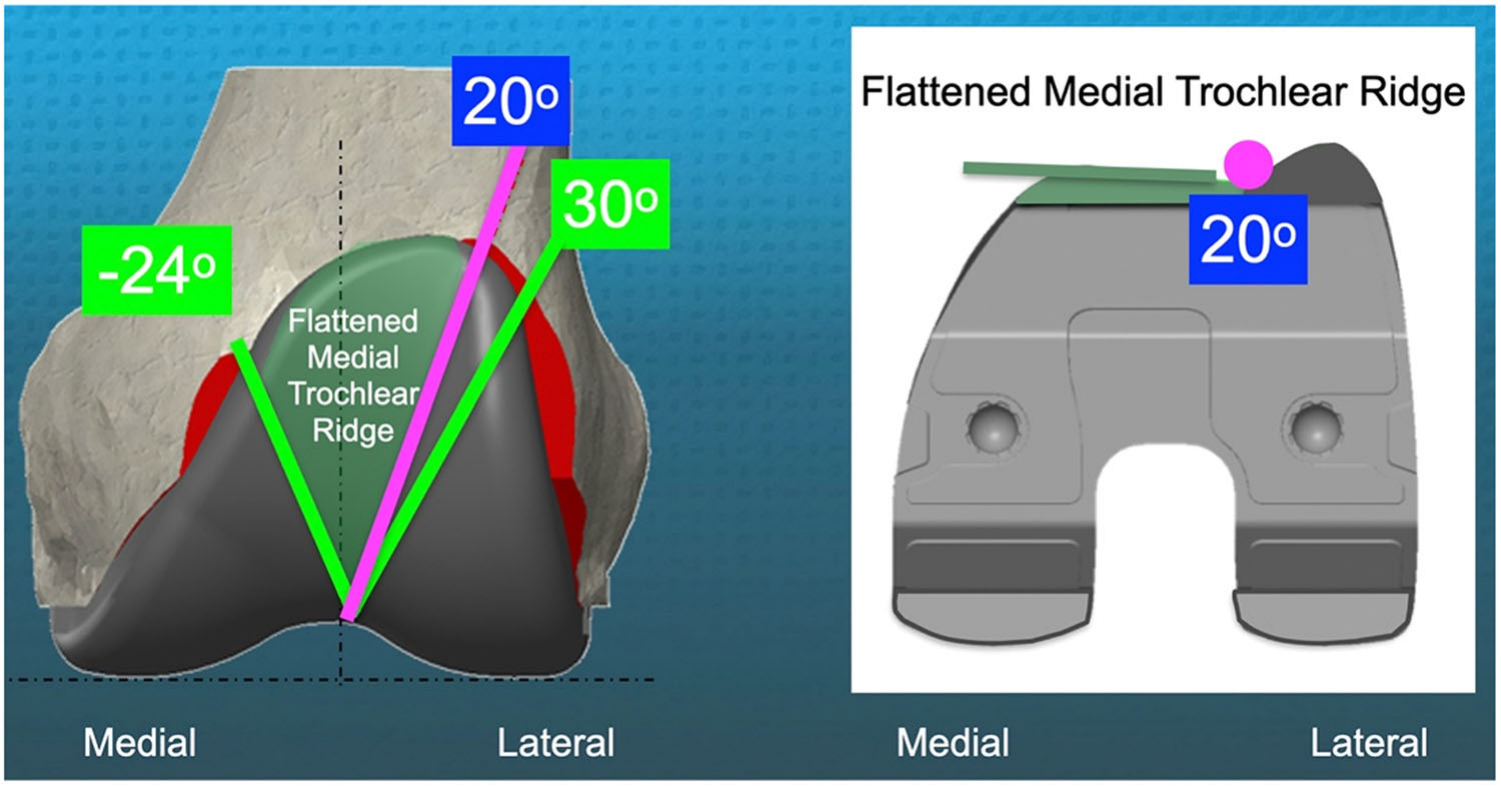
The KA‐optimized femoral component features a prosthetic trochlea with a lateral ridge opening that creates a 20° valgus PTG and a flattened medial ridge (dark green). The 20° lateral ridge compensates for most of the 20% of patients with a groove more valgus than 6°, thus reducing the risk of lateral misalignment of the QLOP outside the groove. Flattening the medial trochlear ridge addresses the needs of 80% of patients with a more varus groove than 6° valgus [41]. KA, kinematic alignment; PTG, prosthetic trochlear groove; QLOP, quadriceps' line of pull.

In November 2021, a new femoral component was introduced featuring a lateral ridge opening that creates a 20° valgus prosthetic trochlear groove (PTG) and a flattened medial ridge (Figure 2). The PTG was increased from 6° to 20° to open the lateral ridge and minimize lateral misalignment of the quadriceps' line of pull (QLOP), thereby restoring a FJS comparable to total hip arthroplasty [18, 19, 21, 43]. The medial ridge was flattened to accommodate the variability in the anterior arthritic groove, which ranges up to −24° varus [41].

The present study of KA TKA aims to describe the deviations in the heights of the medial and lateral trochlear peaks, as well as the I–E tilt, between the prosthetic and patient trochlea. The hypothesis is that correcting for peak under‐stuffing and deviation in I–E tilt is unnecessary, as the PROs are comparable to or better than restorations within a tolerance of ±2 mm.

## MATERIALS AND METHODS

### Patient selection

The study analyzed patients who underwent KA TKA after August 2022. Each patient was treated regardless of the severity of varus or valgus deformity and the degree of flexion contracture. Included were those who had a KA‐optimized femoral component (GMK SpheriKA, Medacta International, www.medacta.com, accessed 5 November 2023), measurements of the heights of the medial and lateral trochlear peaks, a post‐operative long‐leg scanogram, and completed the FJS (100 is *best*, 0 is *worst*), the Oxford Knee Score (OKS) (48 is *best*, 0 is *worst*), and the Knee Injury and Osteoarthritis Outcome Score Joint Replacement (KOOS JR) (100 is *best*, 0 is *worst*) with a minimum follow‐up of 1 year. Individuals with a history of knee fractures treated with open reduction and internal fixation, inflammatory or septic arthritis, or lower extremity neurologic disorders were excluded. Each patient met the following criteria before undergoing KA TKA: fulfilled the Centers for Medicare & Medicaid Services guidelines for medical necessity for TKA and presented with Kellgren–Lawrence Grade III to IV osteoarthritis.

### Outcome measures

On the day of the initial consultation, each patient provided demographic information and completed the OKS and KOOS JR on an iPad. Additionally, the physician assistant recorded knee extension, flexion and alignment deformity using a long‐arm goniometer.

### KA implant design

The features of the KA‐optimized femoral component are detailed in Figure 2. The KA‐optimized tibial component is designed with an asymmetric axial configuration to ensure maximum coverage of the tibial resection. When positioned correctly within the cortical boundary, the anteroposterior axis of the insert aligns parallel to the flexion‐extension plane of the native knee, adhering to KA principles. The insert conformity features a medial socket that allows for a ball‐in‐socket articulation and a lateral flat surface, which supports native internal tibial rotation during flexion. This is essential for restoring the natural patellofemoral kinematics. Additionally, a posterior recess in the insert provides the option for either retaining or excising the posterior cruciate ligament [9, 10, 34, 35].

### Surgical technique

The surgeon used a mid‐vastus approach and manual instruments to perform the KA TKA with PCL retention. Calliper measurements of the bone resections confirmed that the femoral and tibial components resurfaced the pre‐arthritic knee, achieving a reported accuracy of resurfacing the pre‐arthritic knee within 0 ± 0.5 mm, which is essential for optimizing post‐operative OKS and FJS [39]. This accuracy surpasses that of robotics, and there is a negligible learning curve for inexperienced surgeons [12, 17, 31, 44]. The size of the femoral component was chosen to match the maximum width of the distal femur. It was reduced when the anterior gap between the prosthetic trochlea and the anterior femoral resection exceeded 2 mm. The flexion‐extension of the femoral component was established within 1 ± 2° of the sagittal femoral anatomic axis by centring an intraosseous positioning rod in the distal femoral diaphysis [11]. Whenever the inventory included the size and sidedness of the patient's femoral component, the KA‐optimized femoral component was implanted instead of the traditional 6° valgus version designed for MA.

Three checks were performed to ensure the knee was balanced and the appropriate insert thickness was selected. A balanced TKA is defined by restoring the native forces in the medial and lateral tibial compartments and ligament laxities [42, 46]. The varus‐valgus orientation of the tibial resection was carefully adjusted to create a tight rectangular extension space using a spacer block. The slope of the tibial component was restored within ±2° of pre‐arthritic slope by inserting an angel wing through the medial side of the saw slot on the tibial resection guide, placing it ‘over‐the‐top’ of the medial tibial plateau, and adjusting it until the angel wing touches both the anterior and posterior rims of the medial tibial plateau (Dr Stefano Campi, personal communication 27 May 2025). The optimal thickness for the insert allows full knee extension and maximizes internal tibial orientation at 90° of flexion, as measured with an insert goniometer, without causing loss of knee extension and anterior lift‐off from the baseplate [30, 33]. It should be noted that using an insert that is 2 mm thicker than optimal results in doubling the forces in the medial and lateral tibial compartments [46].

The patella was resurfaced using a freehand resection technique, which creates a symmetric resection, in contrast to the use of cutting guides [3, 4]. A broad fan‐shaped saw initiated the osteotomy at the inferior pole of the patella, just posterior to the insertion of the patellar tendon. It was carried proximally, behind the insertion of the quadriceps tendon. A 10‐mm‐thick, anatomically shaped patellar component was implanted. This technique restores the intraoperative patellar thickness within 1 mm in 91% of TKAs [27].

### Calliper measurement of trochlear peaks

After completing the anterior femoral resection, the surgeon measured the heights of the medial and lateral trochlear peaks within ±0.5 mm. A controlled and blinded trial indicates that the calliper measurement of a bone resection has a precision of 0.2 mm, which is better than the calliper's resolution of 0.5 mm. The technique also shows negligible bias or systematic error, along with excellent intra‐class correlation coefficients for repeatability and reproducibility, exceeding 0.95 [44]. An additional millimetre was added to this measurement to account for the bone loss due to the saw blade's kerf. The methods for determining the deviation in peak height and I–E tilt in the prosthetic trochlear plane are detailed in Figure 1.

### Post‐operative care and follow‐up

**Figure 3:**
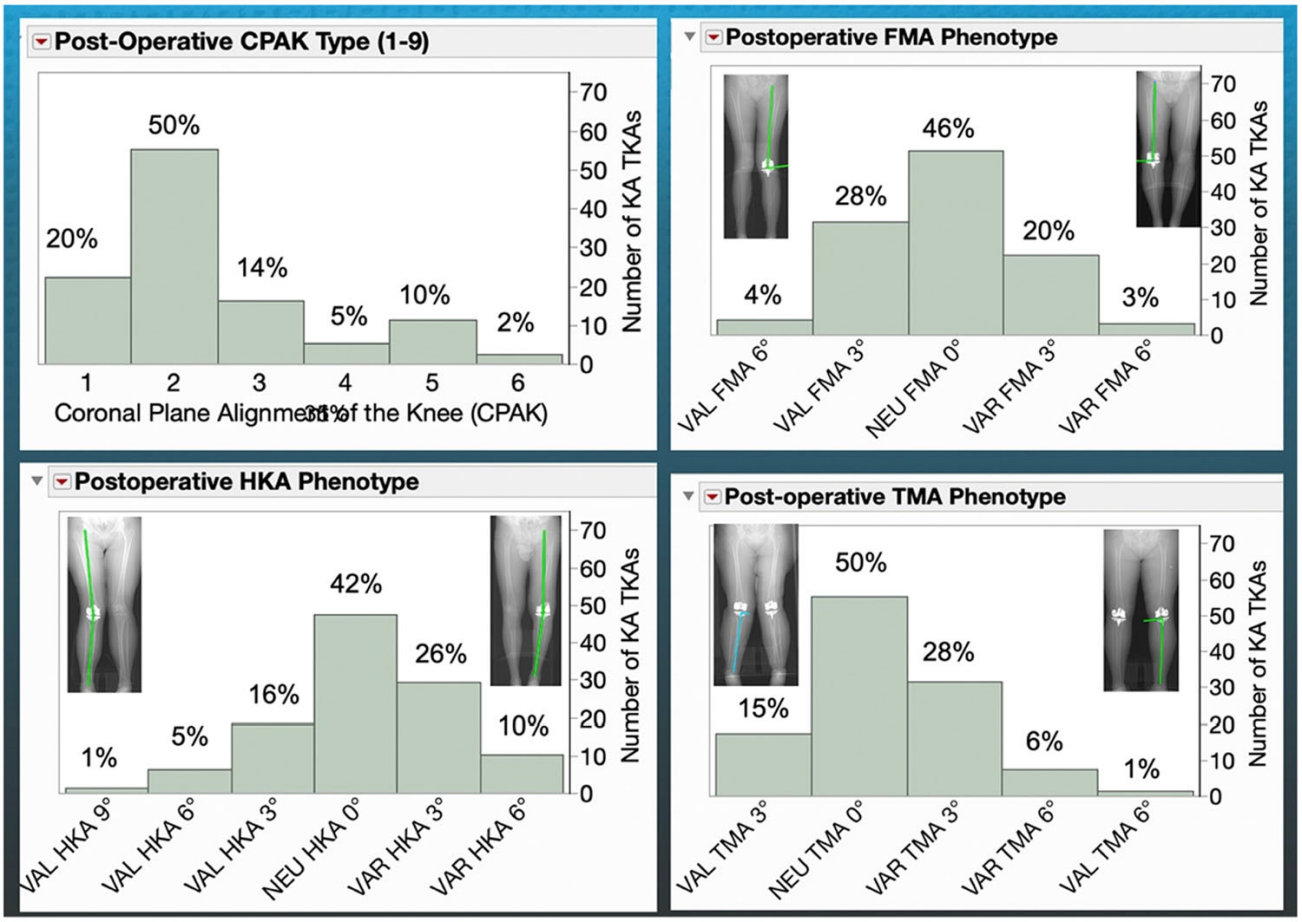
The distributions presented illustrate the post‐operative percentages of KA TKAs categorized by CPAK type and classified according to functional phenotypes: HKA, FMA and TMA. These classifications follow the ranges reported for normal limbs [14, 16, 28]. The scanogram located above each column highlights the most aberrant alignment [15]. However, according to the principles of KA, no component or limb alignment is considered aberrant, as they aim to restore the pre‐arthritic knee and remain within 2° of the alignment of the contralateral side [32]. FMA, femoral mechanical angle; HKA, hip–knee–ankle angle; KA, kinematic alignment; TKA, total knee arthroplasty; TMA, tibial mechanical angle.

On the day of discharge, each patient underwent an A‐P, non‐weight‐bearing, long‐leg scanogram of both legs, performed by a CT scanner with a radiation dose of 0.5 mSv, which is less than that of a long‐leg radiograph [21]. One hundred seven out of 115 patients had a correctly oriented limb for measurement without evidence of hip or ankle arthroplasty or femoral and tibial malunion. The following parameters were measured and then further reported in distributions: the hip–knee–ankle angle (HKA), femoral mechanical angle (FMA), tibial mechanical angle (TMA) and Coronal Plane Alignment of the Knee (CPAK) and HKA, FMA and TMA phenotypes using reported protocols [14, 15, 16, 28] (Table 1 and Figure 3). Each patient was discharged home on the day of surgery and performed exercises at home without the guidance of a physical therapist [1].

**Table 1:** The table summarizes the patient's pre‐operative characteristics and patient‐reported outcome scores, and the post‐operative radiographic long‐leg alignment.

Pre‐operative characteristics	
Number of KA TKAs	115
Age	70 ± 9 years
Sex	58 females, 57 males
Body mass index	30 ± 5 kg/m^2^
Knee extension	7 ± 7°
Knee flexion	10 ± 10°
Clinical varus (−) valgus (+) deformity	−1° varus ± 11° (−20° to 22°)
Pre‐operative patient‐reported outcome measures	
Oxford Knee Score (48 is *best*, 0 is *worst*)	25 ± 9 points
Knee Society Score Knee (100 is *best*, 0 is *worst*)	58 ± 29 points
Knee Society Score Functional (100 is *best*, 0 is *worst*)	61 ± 19 points
KOOS JR	52 ± 13 points
Post‐operative radiographic alignment	
Hip–knee–ankle angle	0 ± 3° (−6° varus to 9° valgus)
Distal lateral femoral angle	3° valgus ± 3° (−10° varus to 14° valgus)
Proximal tibial angle	−4° varus ± 2° (−10° varus to 2° valgus)

*Note*: Reported as mean ± standard deviation.

Abbreviations: KA, kinematic alignment; KOOS JR, Knee Injury and Osteoarthritis Outcome Score for Joint Replacement; TKA, total knee arthroplasty.

Patients received an electronic questionnaire at least 12 months after follow‐up. The study included participants who completed the FJS (100 is *best*, 0 is *worst*), KOOS JR (100 is *best*, 0 is *worst*) and OKS (48 is *best*, 0 is *worst*).

### Ethical considerations

The Advarra Institutional Review Board (www.advarra.com) provided an exempt determination (Pro00084682) for a retrospective analysis of de‐identified patient data obtained from a prospectively archived records database.

### Statistical analysis

A sample size calculation was performed using a free online equivalence trial calculator for three PROs: FJS, KOOS JR and OKS (‘Sealed Envelope’, https://www.sealedenvelope.com/power/continuous-equivalence/, last accessed 1 November 2024). The significance level was set at 0.05, and the power (1 − *β*) was established at 90%. The equivalence limits (*d*) were defined based on the reported minimally clinically important differences for the effect size (*d*). The reported values for (*d*) were as follows: 14 points for the FJS [6], 18 points for the KOOS JR [26] and 5 points for the OKS [5], as detailed in Table 2. Therefore, the minimum sample size was 90 patients.

**Table 2:** The table summarizes the power analysis results for each patient‐reported outcome score and includes the standard deviation, the difference to detect and the sample size.

Dependent variable	Standard deviation of the outcome	Difference to detect, *d*	Total sample size
Patient‐reported outcome scores			
Forgotten Joint Score (Clement 2021) [6]	20	14	90
KOOS JR (Kuo 2020) [26]	18	18	44
Oxford Knee Score (Clement 2014) [5]	6	5	64

Abbreviation: KOOS JR, Knee Injury and Osteoarthritis Outcome Score for Joint Replacement.

Statistical software was used to calculate the mean and standard deviation (SD) for dependent variables that followed a normal distribution (JMP Pro, 18.0.1, http://www.jmp.com, accessed on 29 December 2024). A Student's *t*‐test was employed to assess the significance of differences. For variables that did not meet this criterion, as determined by a goodness‐of‐fit test, the median and interquartile range (IQR) were calculated. Equivalence analyses were used to test the null hypothesis that the FJS, KOOS JR and OKS were comparable for three categories of deviation in height of the peak: restored within ±2 mm, under‐stuffed greater than 2 mm and overstuffed greater than 2 mm. Separate analyses were done for medial and lateral peaks. An equivalence analysis also tested the null hypothesis that the PROs for three categories of the tilt of the trochlear plane: restored within ±2 mm, internal tilt greater than 2 mm and external tilt greater than 2 mm. The analysis utilized a significance level of 0.05, a confidence of 90%, and the difference to detect (*d*) based on the reported minimal clinically important differences above.

## RESULTS

The analysis involved 115 patients. Table 1 summarizes the preoperative demographics, types of knee deformity, extension and flexion, and PROs at a mean of 22 ± 5 months and post‐operative radiographic findings. Post‐operative CPAK and HKA, FMA and TKA phenotypes are shown in Figure 3.

**Figure 4:**
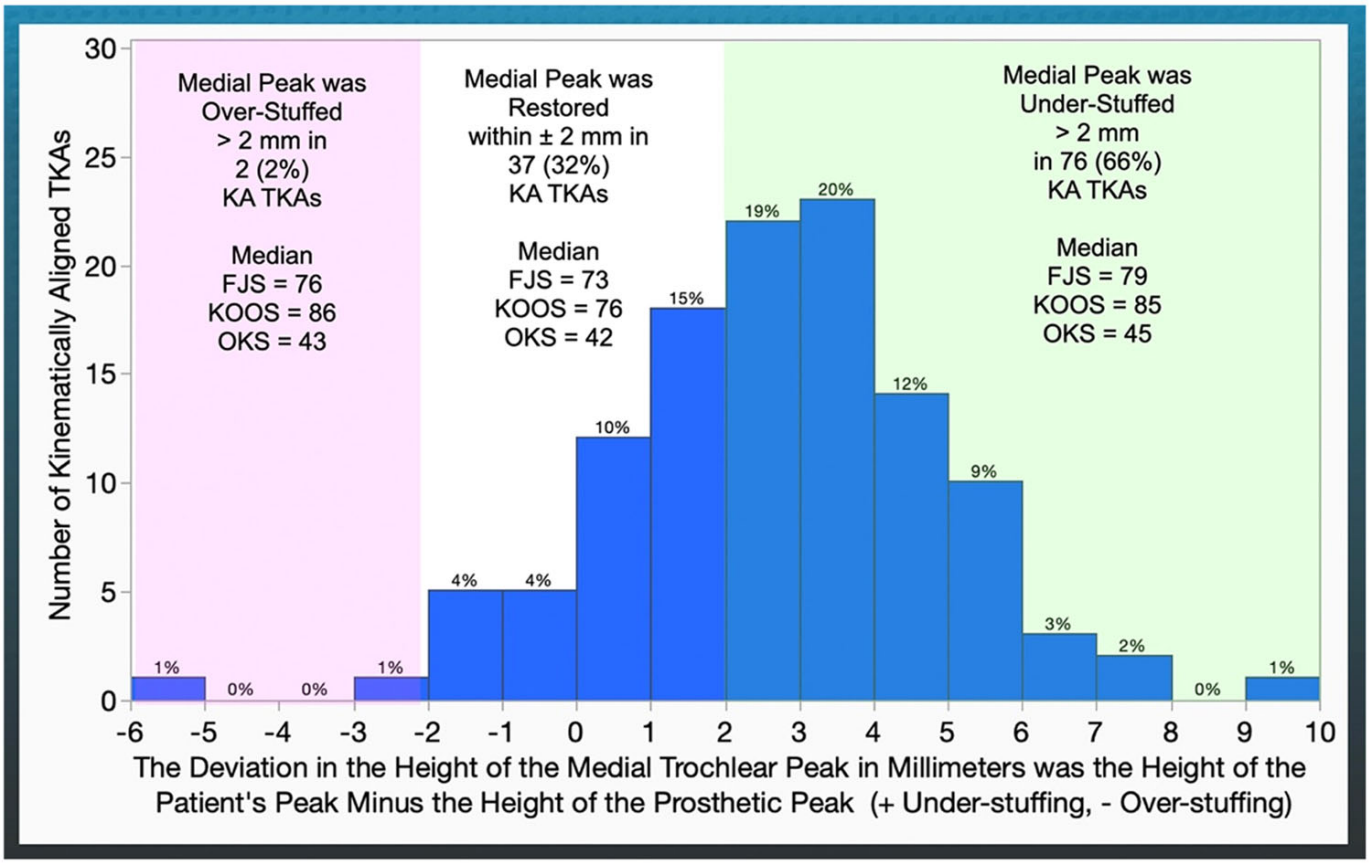
The distribution illustrates the number of KA TKAs with a deviation in the height of the medial trochlear peak relative to the thickness of the prosthetic peak in millimetres. The text on the shaded rectangles describes the proportion of TKAs and the median value for PROs for patients classified into under‐stuffing >2 mm, over‐stuffing >2 mm and restoration within ±2 mm. Note that the highest median PROs are in those patients with under‐stuffing >2 mm. FJS, Forgotten Joint Score; KA, kinematic alignment; KOOS, Knee Injury and Osteoarthritis Outcome Score; OKS, Oxford Knee Score; PRO, patient‐reported outcome; TKA, total knee arthroplasty.

Regarding the medial peak, under‐stuffing was noted in 76 (66%) patients and over‐stuffing in 2 (2%). Compared to restoration within ±2 mm, medial peak under‐stuffing resulted in a non‐equivalent 6‐point higher FJS (*p* = 0.1087) and equivalent but 9‐ and 3‐point higher KOOS (*p* < 0.0001) and OKS (*p* = 0.0020) (Figure 4 and Table 3).

**Table 3:** The table on the deviation of prosthetic medial trochlear peak height displays the results of equivalence analyses comparing the median [interquartile range] of the Forgotten Joint Score (FJS), KOOS JR and Oxford Knee Score (OKS) among patients who are restored within ±2 mm (*n* = 37), under‐stuffed by >2 mm (*n* = 76), or over‐stuffed by >2 mm (*n* = 2) after KA TKA with a 20° KA‐optimized femoral component.

Equivalence teat	Outcomes scores			Difference to detect, *d*	Result of equivalence test
Restored vs. under‐stuffed		Medial peak within ±2 mm	Medial peak under‐stuffed >2 mm		
	FJS	73 [41–83]	79 [57–92]	14 points	Not equivalent, *p* = 0.1087
	KOOS JR	76 [68–92]	85 [73–100]	18 points	Equivalent, *p* < 0.0001
	OKS	42 [48–46]	45 [40–47]	5 points	Equivalent, *p* = 0.0011
Restored vs. over‐stuffed^a^		Medial peak within ±2 mm	Medial peak over‐stuffed >2 mm		
	FJS	73 [41–83]	76 [60–92]	14 points	Not equivalent, *p* = 0.4493^a^
	KOOS JR	76 [68–92]	86 [71–100]	18 points	Not equivalent, *p* = 0.1317^a^
	OKS	42 [48–46]	43 [39–46]	5 points	Not equivalent, *p* = 0.1728^a^

Abbreviations: KA, kinematic alignment; KOOS JR, Knee Injury and Osteoarthritis Outcome Score for Joint Replacement; OKS, Oxford Knee Score; TKA, total knee arthroplasty.

a The statistical analysis was inadequate for interpretation since only two subjects exhibited medial over‐stuffing.

**Figure 5:**
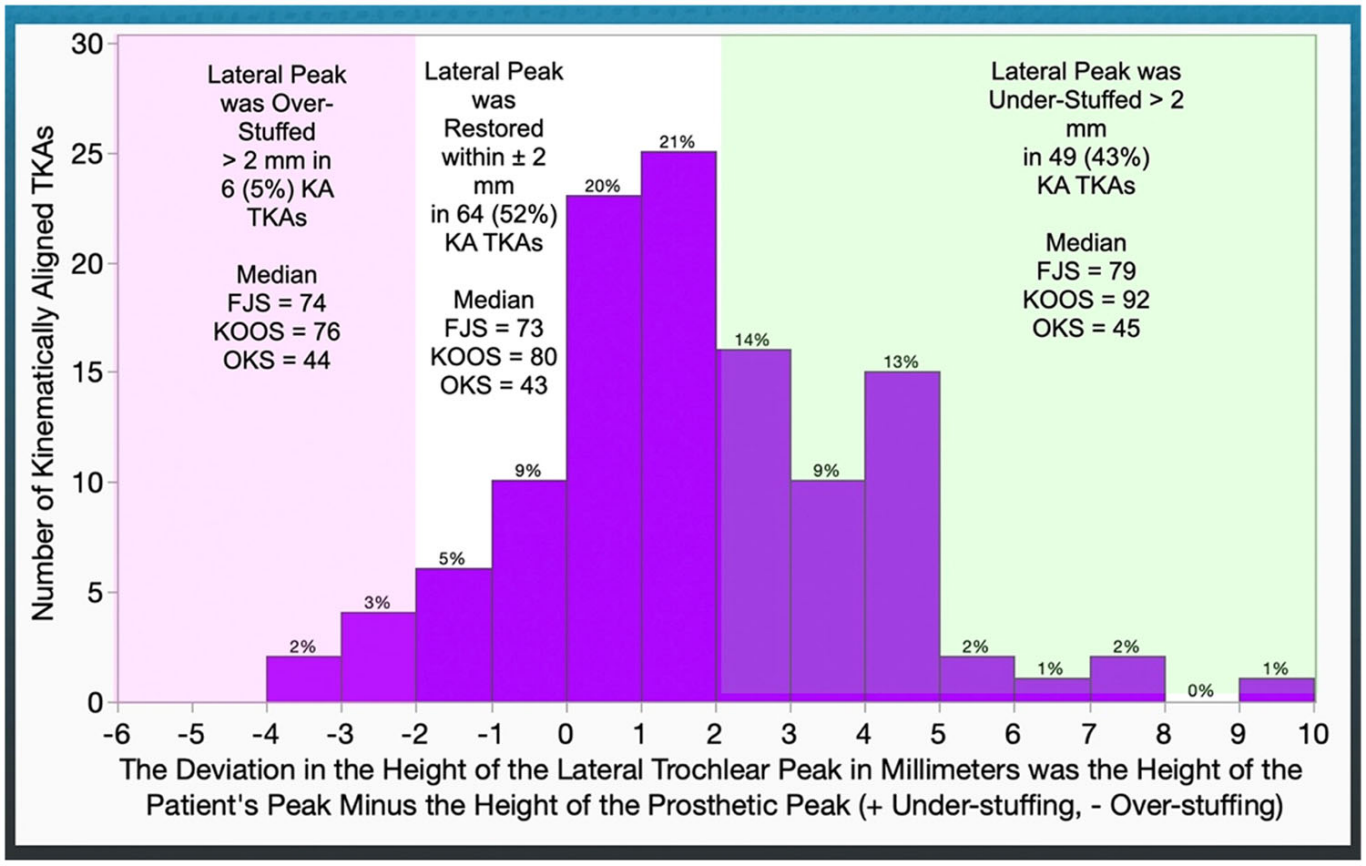
The distribution illustrates the number of KA TKAs with a deviation in the height of the lateral trochlear peak relative to the thickness of the prosthetic peak in millimetres. The text on the shaded rectangles describes the proportion of TKAs and the median value for PROs for patients classified into under‐stuffing >2 mm, over‐stuffing >2 mm and restoration within ±2 mm. Note that the highest median PROs are in those patients with under‐stuffing >2 mm. FJS, Forgotten Joint Score; KA, kinematic alignment; KOOS, Knee Injury and Osteoarthritis Outcome Score; OKS, Oxford Knee Score; PRO, patient‐reported outcome; TKA, total knee arthroplasty.

As for the lateral peak, under‐stuffing was noted in 49 (43%) patients and over‐stuffing in 6 (5%). Lateral peak under‐stuffing yielded equivalent but 6‐, 12‐ and 3‐point higher FJS (*p* = 0.0484), KOOS (*p* < 0.0001) and OKS (*p* < 0.0001) compared to peak restoration (Figure 5 and Table 4).

**Table 4:** The table on the deviation of prosthetic lateral trochlear peak height displays the results of equivalence analyses comparing the median [interquartile range] of the Forgotten Joint Score (FJS), KOOS JR and Oxford Knee Score (OKS) among patients who are restored within ±2 mm (*n* = 60), under‐stuffed by >2 mm (*n* = 49) or over‐stuffed by >2 mm (*n* = 6) after KA TKA with a 20° KA‐optimized femoral component.

Equivalence teat	Outcomes scores			Difference to detect, *d*	Result of equivalence test
Restored vs. under‐stuffed		Lateral peak within ±2 mm	Lateral peak under‐stuffed >2 mm		
	FJS	73 [48–85]	79 [57–92]	14 points	Equivalent, *p* = 0.0408
	KOOS JR	80 [68–98]	92 [76–100]	18 points	Equivalent, *p* < 0.0001
	OKS	43 [39–46]	45 [43–47]	5 points	Equivalent, *p* = 0.0020
Restored vs over‐stuffed^a^		Lateral peak within ±2 mm	Lateral peak over‐stuffed >2 mm		
	FJS	73 [48–85]	74 [58–94]	14 points	Not equivalent, *p* = 0.2884^a^
	KOOS JR	80 [68–98]	76 [71–89]	18 points	Equivalent, *p* = 0.0036^a^
	OKS	43 [39–46]	44 [39–46]	5 points	Not equivalent, *p* = 0.0569^a^

Abbreviations: KA, kinematic alignment; KOOS JR, Knee Injury and Osteoarthritis Outcome Score for Joint Replacement; OKS, Oxford Knee Score; TKA, total knee arthroplasty.

a The statistical analysis was inadequate for interpretation since only six subjects exhibited lateral over‐stuffing.

**Figure 6:**
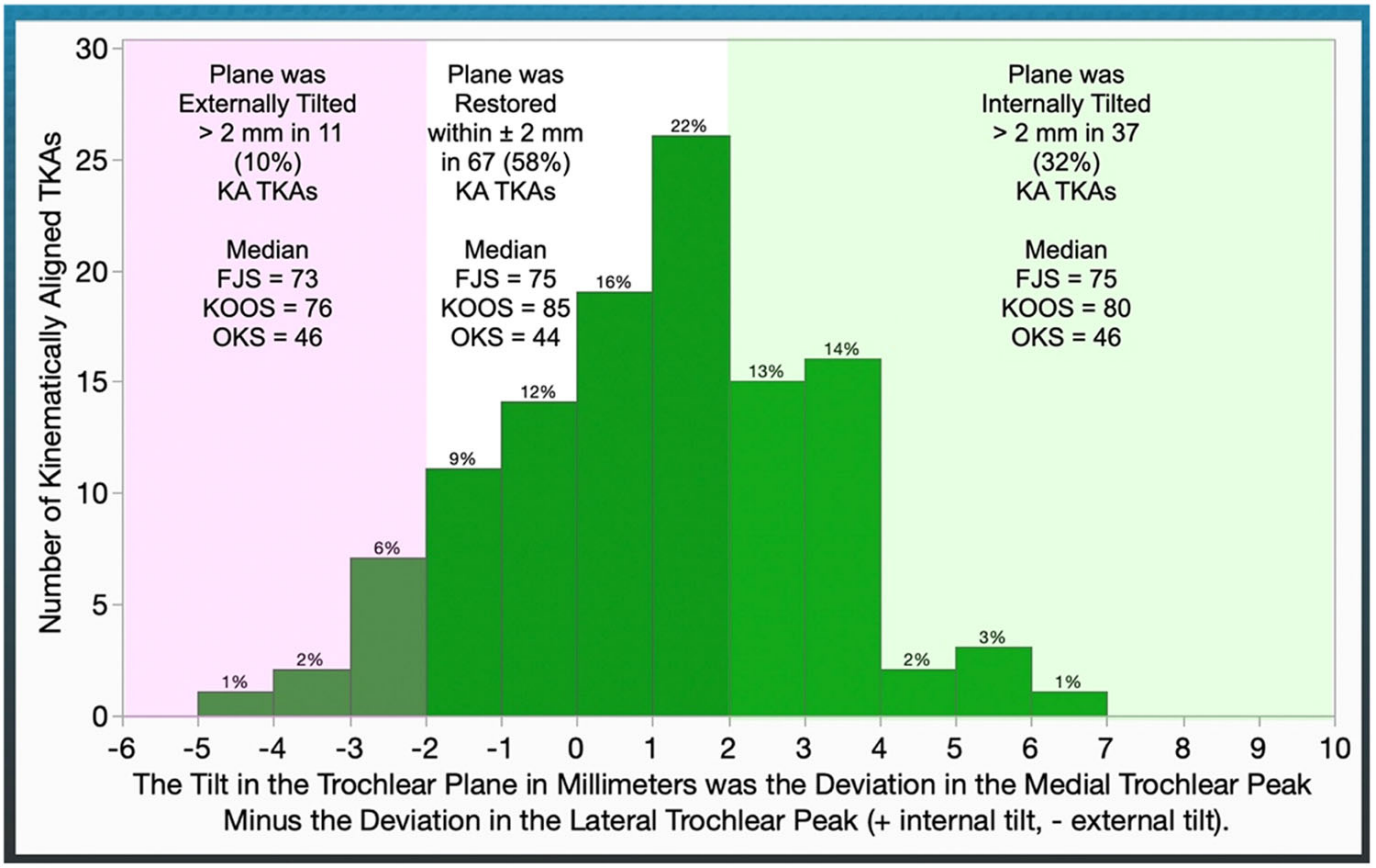
The distribution illustrates the number of KA TKAs with a tilt in the anterior prosthetic trochlear plane relative to the patient's plane. The text on the shaded rectangles describes the proportion of TKAs and the median value for PROs for patients classified as having greater than 2 mm of internal (+) and external (−) tilt, and restoration within ±2 mm. Note that internal–external tilt >2 mm has little effect on PROs. FJS, Forgotten Joint Score; KA, kinematic alignment; KOOS, Knee Injury and Osteoarthritis Outcome Score; OKS, Oxford Knee Score; PRO, patient‐reported outcome; TKA, total knee arthroplasty.

In terms of the tilt of the trochlear plane, internal tilt was noted in 37 (32%) patients and external tilt in 11 (10%). I–E tilt >2 mm has little effect on PROs (Figure 6 and Table 5).

**Table 5:** The table on the tilt of the prosthetic trochlear plane displays the results of equivalence analyses comparing the median [interquartile range] of the Forgotten Joint Score (FJS), KOOS JR and Oxford Knee Score (OKS) among patients who are restored within ±2 mm (*n* = 67), internal tilt >2 mm (*n* = 37) or external tilt by >2 mm (*n* = 11) after KA TKA with a 20° KA‐optimized femoral component.

Equivalence teat	Outcomes scores			Difference to detect, *d*	Result of equivalence test
Restored vs. under‐stuffed		Tilt within ±2 mm	Internal tilt >2 mm		
	FJS	75 [48–88]	75 [59–96]	14 points	Equivalent, *p* = 0.0181
	KOOS JR	85 [71–100]	80 [74–100]	18 points	Equivalent, *p* < 0.0001
	OKS	44 [40–46]	46 [41–47]	5 points	Equivalent, *p* = 0.0003
Restored vs. over‐stuffed		Tilt within ±2 mm	External tilt >2 mm		
	FJS	75 [48–88]	73 [25–88]	14 points	Not equivalent, *p* = 0.1983
	KOOS JR	85 [71–100]	76 [68–100]	18 points	Equivalent, *p* = 0.0063
	OKS	44 [40–46]	46 [37–48]	5 points	Not equivalent, *p* = 0.0766

Abbreviations: KA, kinematic alignment; KOOS JR, Knee Injury and Osteoarthritis Outcome Score for Joint Replacement; OKS, Oxford Knee Score; TKA, total knee arthroplasty.

## DISCUSSION

The most significant findings of this study on KA TKA, which evaluated a KA‐optimized femoral component featuring a lateral ridge opening that creates a 20° valgus PTG and a flattened medial ridge, were as follows: (1) Under‐stuffing of the medial and lateral trochlear peaks is common; (2) under‐stuffing results in equivalent or higher PROs than the restoration of peak height; and (3) internal–external tilt of the prosthetic trochlear plane of >2 mm had no adverse effect on PROs.

Under‐stuffing the medial and lateral peaks by >2 mm may be preferable to restoration within ±2 mm, as it is associated with higher FJS, KOOS JR and OKS—an unexpected finding. One explanation for the increase in PROs from under‐stuffing peak height >2 mm is that it compensates for over‐stuffing above the native trochlea caused by the prosthesis's proximal overreach, which averages 17 mm (range, 7–32 mm) [29]. A second explanation is that the flattening of the medial trochlear ridge compensates for over‐stuffing in patients where a more prominent medial ridge overlies a varus anterior arthritic trochlear groove, with an upper limit of −24° [41].

Under‐stuffing the height of the trochlear peaks induces negligible changes in the patella moment arm once the knee is flexed because the distance between the condyles of the femoral component, which restores the pre‐arthritic articular surface, and the patella flexion‐extension axis determines the moment arm length instead of the height of the trochlear peaks. This means that the moment arm along the 45–60° arc, where the moment is largest, is unaffected by changes in the peak height in the anterior trochlea, which explains why there were no adverse effects on PROs [20, 47].

Surgeons who favour the calliper resection technique might speculate that the freehand flush resection technique systematically under‐resected the patella in the present study. Comparatively, the calliper resection technique may lead to over‐stuffing of the patellar portion of the anterior compartment in 44% of TKAs, necessitating under‐stuffing of the trochlear portion as a compensatory measure [13]. Fortunately, KA with a KA‐optimized prosthetic trochlea featuring a lateral ridge opening that creates a 20° valgus PTG and a flattened medial ridge, provides the necessary compensation by under‐stuffing the femoral side of the anterior compartment. Thus, the study's primary finding—that >2 mm of trochlear peak under‐stuffing leads to superior PROs—still applies to those who utilize the calliper patella resection technique.

Although there were too few patients with medial (2%) and lateral (5%) over‐stuffing >2 mm to permit statistical analysis, the PROs were not lower than those of restoration. This finding contrasts with a lower Knee Society Function score and knee flexion at 1 year, which was reported as over‐stuffing after FA TKA [39]. One possible explanation for this difference is that FA can over‐resect the posteromedial posterior femoral articular surface by >1 mm when externally rotating the femoral component, which decreases the FJS score by 35 points and the OKS score by 9 points [39]. In contrast, the KA technique does not externally rotate the femoral component because it restores the pre‐arthritic posterior femoral joint line in all patients.

The −4 mm internal tilt combined with a 6 mm external tilt of the prosthetic trochlear plane relative to the patient's trochlear plane did not negatively impact PROs. This finding is consistent with a study on KA TKA, which examined post‐operative radiographic factors related to patellofemoral tracking. These factors included the patella tilt angle (ranging from 10° internal to 14° external), lateral patella translation, and lateral under‐coverage of the anterior femoral resection. Because none of these factors predicted clinical outcome scores or patient dissatisfaction, they were not measured again in the current study [8].

Several limitations should be acknowledged. First, all procedures were performed by a single, experienced surgeon specializing in knee arthroplasty (KA). This may limit the generalizability of the results to surgeons with varying levels of experience. However, performing KA with manual instruments offers significantly greater accuracy in resecting the distal and posterior femur than robotic methods and patient‐specific instrumentation, with a negligible learning curve [31, 44]. Second, the study did not obtain the Kujala score, which is designed to assess physical symptoms and limitations in the patellofemoral joint. This omission is unlikely to affect the study's findings because changes in the Kujala score parallel those of the OKS and KOOS scores. Additionally, the Kujala score is expected to have a high ‘ceiling effect’ similar to the 14% for the OKS and 24% for the KOOS, which limits its ability to differentiate subtle variations in outcomes [7, 38].

## CONCLUSION

When performing KA TKA, a KA‐optimized femoral component provides a patient‐specific solution that addresses variations in the anterior arthritic trochlear groove, ranging from −24° varus to 30° valgus [41]. Surgeons should resist the temptation to reduce the under‐stuffing of the medial and lateral trochlear peaks and strictly adhere to the principle of resurfacing the pre‐arthritic femur because the PROs are equivalent to or higher than those of peak restoration. Additionally, anteriorizing the posteromedial articular surface by more than 1 mm results in poor PROs and potentially leads to tibiofemoral instability [39].

## AUTHOR CONTRIBUTIONS

All authors contributed to the study's conception, design, preparation of materials, data collection, analysis and writing. All authors also read and approved the final manuscript.

## CONFLICTS OF INTEREST STATEMENT

Stephen M. Howell and Alexander J. Nedopil receive consulting fees and royalties from Medacta International (www.medacta.com). Maury L. Hull receives research support from Medacta International (www.medacta.com). The remaining authors declare no conflicts of interest.

## ETHICS STATEMENT

The institutional review board approved this study, specifically the retrospective analysis of de‐identified data (Pro00084429) obtained from a prospectively archived patient records and radiographs database.

## Data Availability

The data sets used and/or analyzed during the current study are available from the corresponding author upon reasonable request.
